# The impact of smoking different tobacco types on the subgingival microbiome and periodontal health: a pilot study

**DOI:** 10.1038/s41598-020-80937-3

**Published:** 2021-01-13

**Authors:** Sausan Al Kawas, Farah Al-Marzooq, Betul Rahman, Jenni A. Shearston, Hiba Saad, Dalenda Benzina, Michael Weitzman

**Affiliations:** 1 Department of Oral and Craniofacial Health Sciences, College of Dental Medicine, University of Sharjah, Sharjah, UAE; 2 Sharjah Institute for Medical Research, University of Sharjah, Sharjah, UAE; 3 Department of Medical Microbiology and Immunology, College of Medicine and Health Sciences, UAE University, P.O. Box 15551, Al Ain, UAE; 4 Department of Preventive and Restorative Dentistry, College of Dental Medicine, University of Sharjah, Sharjah, UAE; 5 Department of Pediatrics, School of Medicine, New York University, New York, USA; 6 New York University Abu Dhabi, Al Ain, UAE; 7 Department of Environmental Health Sciences, Mailman School of Public Health, Columbia University, New York, USA; 8 Department of Environmental Medicine, School of Medicine, New York University, New York, USA; 9 College of Global Public Health, New York University, New York, USA

**Keywords:** Microbiology, Molecular biology, Health care, Medical research, Risk factors

## Abstract

Smoking is a risk factor for periodontal disease, and a cause of oral microbiome dysbiosis. While this has been evaluated for traditional cigarette smoking, there is limited research on the effect of other tobacco types on the oral microbiome. This study investigates subgingival microbiome composition in smokers of different tobacco types and their effect on periodontal health. Subgingival plaques were collected from 40 individuals, including smokers of either cigarettes, medwakh, or shisha, and non-smokers seeking dental treatment at the University Dental Hospital in Sharjah, United Arab Emirates. The entire (~ 1500 bp) 16S rRNA bacterial gene was fully amplified and sequenced using Oxford Nanopore technology. Subjects were compared for the relative abundance and diversity of subgingival microbiota, considering smoking and periodontal condition. The relative abundances of several pathogens were significantly higher among smokers, such as *Prevotella denticola* and *Treponema* sp. *OMZ 838* in medwakh smokers, *Streptococcus mutans* and *Veillonella dispar* in cigarette smokers*, Streptococcus sanguinis* and *Tannerella forsythia* in shisha smokers. Subgingival microbiome of smokers was altered even in subjects with no or mild periodontitis, probably making them more prone to severe periodontal diseases. Microbiome profiling can be a useful tool for periodontal risk assessment. Further studies are recommended to investigate the impact of tobacco cessation on periodontal disease progression and oral microbiome.

## Introduction

Periodontal disease is considered the most common chronic inflammatory disease of the oral cavity and a major cause of tooth loss in adult population worldwide^1,2^. It encompasses polymicrobial infectious disorders affecting the tooth-supporting tissues, including inflammation of the gingiva (gingivitis), alveolar bone, and periodontal ligament (periodontitis), which are risk factors for tooth loss^3^. Periodontal disease is a prototype of local destructive, chronic, low‐grade inflammation, and has been linked to different systemic diseases such as cardiovascular disease, diabetes mellitus and cancer in the mouth, gastrointestinal tract, lung, and pancreas^4^.

Periodontal inflammation is driven by the existence of microbial complexes in the subgingival plaque^5^. It is believed that certain periodontal pathogens are responsible for the initiation and further development of periodontal disease, such as *Tannerella forsythia*, *Treponema denticola* and *Porphyromonas gingivalis*^6^. In addition, genetic and environmental factors might increase the susceptibility of the host to periodontal disease^7^.

Smoking is among the major risk factors predisposing individuals to oral diseases such as periodontitis and cancer^8^. Smoking has been shown to increase the relative risk of destructive periodontal disease by at least five to sixfold^4^. Several studies have reported that smokers have a more severe form of the disease, reflected by increased loss of attachment, increased gingival recession, and more rapid development and progression of periodontal inflammation^9^. Smoking increases the prevalence and severity of periodontal destruction by altering the host immune response, making the oral cavity more vulnerable to propagation of pathogenic bacterial species^10^. Several studies have linked smoking to dysbiosis of the oral microbiota, increasing the risk of periodontal disease^11–13^.

The oral microbiome plays key roles in human health and may have a role in many diseases, including periodontal disease^14–17^. Tobacco smoking affects the microbial ecology of the oral cavity through immunosuppression, oxygen deprivation, antibiotic effects, and other possible mechanisms^8^. Loss of beneficial oral species due to smoking can lead to pathogen colonization and ultimately to progression of periodontitis^18^. Studies have found that the subgingival microbial profiles of smokers are highly diverse, pathogen-rich, anaerobic, and are more closely aligned with a disease-associated community, even in clinically healthy individuals^11,12,19^.

Alternative forms of tobacco smoking such as shisha via waterpipe use is rapidly increasing and spreading worldwide^20–22^. Shisha use is increasing globally due to its use in social settings, pleasant flavor, and the misperception of harm reduction by the passing of smoke through water^23,24^. Dokha is another alternative form of tobacco consisting of tobacco mixed with herbs and other substances, smoked out of a narrow pipe called medwakh. It is available in different strengths from mild to strong, and is typically smoked by taking a few inhalations in a single sitting, an average of 12 times per day^25^. Dokha is one of the most rapidly spreading alternative tobacco products in the Middle East and North Africa (MENA) region^26^. It has been reported that medwakh smoking is the second most common form of tobacco used among men in Abu Dhabi in the United Arab Emirates (UAE), after cigarette smoking^27^.

Several studies have compared the subgingival microbiome between cigarettes smokers and non-smoker individuals with chronic moderate periodontitis^28–30^; however, the effect of smoking different tobacco types on the subgingival microbiome has not been reported. To the best of our knowledge, this is the first study evaluating the relationship between medwakh use, the subgingival oral microbiome, and periodontal disease. The aim of this study is to investigate the effect of smoking different tobacco types (including medwakh, shisha and cigarettes) on the composition of the subgingival microbiome in patients with or without periodontal disease, in order to gain a better understanding of the pathogenesis of periodontitis in smokers and to explore the possibility of predicting the disease before its occurrence (or at early stages).

## Methods

### Study population

The study protocol was approved by the Research Ethics Committee at the University of Sharjah, UAE (REC-18-10-23-01), in compliance with the national and international standards including Helsinki declaration. All methods and procedures were performed in accordance with the relevant guidelines and regulations. In the period between October 2018 and October 2019, patients seeking dental treatment at the University Dental Hospital Sharjah, Sharjah, UAE were invited to participate in the study. Patients who agreed to participate in the study and signed the informed consent were recruited based on the following inclusion criteria: being an adult aged 18–60 year, having at least 10 teeth, and either being a non-smoker or user of only one type of tobacco (cigarettes, shisha, or medwakh). Patients who were currently receiving orthodontic treatment and those who had any periodontal treatment, antibiotics, or steroid therapy in the last 3 months were excluded. Female patients who were pregnant and medically compromised patients (patients with chronic systemic diseases, cardiovascular diseases, or diabetes) were also excluded. In total, 40 patients were recruited into four groups (n = 10 each): non-smokers, cigarette-only smokers, shisha-only smokers, or medwakh-only smokers.

We conducted a comprehensive oral exam and assessment of oral microbiome community composition and taxon abundance, by bacterial 16S rRNA gene sequencing of subgingival plaque for these 40 individuals.

### Periodontal assessment

Complete periodontal examination was performed by a trained dental professional to assess gingival inflammation, i.e. bleeding on probing (BOP), probing pocket depth (PPD) and clinical attachment level (CAL) on six anatomical sites for each tooth^31^. Periodontal disease diagnosis was assigned to participants according to periodontitis definition accepted in the 2017 world workshop. A patient was considered as a periodontitis case when interdental CAL was detectable at ≥ 2 non-adjacent teeth; or buccal or lingual CAL ≥ 3 mm with > 3 mm pocket depth detectable at ≥ 2 teeth^32^. According to the classification of AAP/EFP, when interdental CAL at site of greatest loss was 1–2 mm, case was described as Stage I periodontitis (mild or initial periodontitis) with no tooth loss due to periodontitis and maximum probing depth was ≤ 4 mm. When interdental CAL at site of greatest loss was 3–4 mm, no tooth loss due to periodontitis and maximum probing depth was ≤ 5 mm, case was defined as Stage II periodontitis (moderate periodontitis). When interdental CAL at the site of greatest loss was ≥ 5 mm with ≤ 4 teeth loss due to periodontitis and probing depth ≥ 6 mm, case was defined as Stage III periodontitis (severe periodontitis). When case has ≥ 5 teeth lost due to periodontitis in addition to the Stage III criteria and needs complex rehabilitation, it was defined as Stage IV periodontitis (advanced periodontitis)^32^. Based on the criteria above, we grouped the participants into two groups, including subjects with no or mild periodontitis (no or stage I), and subjects with moderate to severe periodontitis (Stage II to Stage III).

### Specimen collection

Subgingival plaque samples were collected from all participants at the same time of day (in the afternoon, approximately 5–7 h after tooth brushing). Samples from periodontally healthy patients were collected by passing paper points across each gingival sulcus and pooled from eight teeth in quadrants 1 and 3 (incisor, canine, premolar, and molar). In periodontitis patients, subgingival samples were collected and pooled from the deepest PPD sites in each quadrant (eight non-adjacent proximal sites)^33^. Samples were collected from periodontitis patients by inserting 16 sterile endodontic paper points (size 30; two paper points per site) into the gingival sulci or periodontal pocket, for 10 s, following isolation and supragingival plaque removal. Samples were placed in 1.5 ml sterile tubes with 300 μl of phosphate buffer, placed on dry ice, then transferred to − 80 °C freezer until further analysis^34^.

### Nanopore sequencing of 16S rRNA gene from the subgingival plaques

Microbiota were identified using third-generation sequencing with Nanopore technology, on a MinION device (Oxford Nanopore Technologies, UK). DNA was isolated from each sample using the Epicentre MasterPure DNA Purification Kit (Epicenter, USA), according to the manufacturer’s recommended protocol. The quality and quantity of the extracted DNA was assessed using a nanodrop (Colibri Microvolume Spectrometer; Titertek-Berthold, Germany).

The entire (~ 1500 bp) 16S rRNA gene was fully amplified using the 16S Barcoding Kit (SQK‐RAB204; Oxford Nanopore Technologies, Oxford, UK) and LongAmp Taq 2× Master Mix (New England Biolabs, UK) with 1 µg of input DNA per sample. Amplification was performed using an Applied Biosystems Veriti Thermal Cycler (Thermo Scientific, USA)^35^. PCR products were purified using AMPure XP (Beckman Coulter, USA) and quantified by fluorometer Qubit 4 (Thermo Scientific, US). Equimolar amounts of the amplification products were pooled together in a single tube. A total of 100 ng DNA of the pooled sample was used for library preparation, and MinION sequencing was performed using R9.4 flow cells (Oxford Nanopore Technologies, UK) according to the manufacturer's instructions^35^. MinKNOW version 2.0 (Oxford Nanopore Technologies, UK) was used for live basecalling and data acquisition^36^. Guppy v3.4.4 was used to convert raw signal data into FASTQ format, demultiplexing, and removal of nanopore and adaptor sequences^37^. FASTQ files were analyzed on the Nanopore EPI2ME platform with a default minimum Q score of 7. Preliminary bacterial identification was done via ‘What’s in my Pot?’ (WIMP) workflow provided by Oxford Nanopore Technologies, UK^38^. Reads assigned to all targets were re-analyzed by Kraken taxonomic sequence classification system (version 2.0.8-beta)^39^ using Partek Genomics Suite software, version 7.0 (Copyright 2020; Partek Inc., St. Louis, MO, USA). The numbers of reads assigned per taxon were counted and the relative abundance of reads per taxon were used for separate downstream analysis.

### Statistical analyses

Continuous variables were presented using mean ± SD. Statistical comparison of clinical, demographic, microbiota relative abundance, and alpha diversity were made using the Kruskal–Wallis test to compare samples grouped based on tobacco types smoked, and the Mann–Whitney U test to compare samples grouped according to periodontal condition. Correlations between relative abundance of taxa and clinical parameters of periodontal disease were calculated using Spearman correlation coefficients (SPSS software, version 20). All statistical tests were two-sided. A p-value < 0.05 was considered statistically significant.

Venn diagrams were generated to show the shared and unique operational taxonomic units (OTU) among groups, based on the occurrence of OTUs in a group regardless of their relative abundance using the Venny bioinformatic tool (version 2.1)^40^. Shared taxa (genera and species) present in all four groups were defined as the core microbiome^36^. For statistical comparison of the relative abundance, only taxa that were detected at ≥ 1% abundance in at least one sample were considered. For the genera, relative abundance values were transformed into log2 values, then the mean value of each smoking group was normalized against the non-smokers as a control group to calculate log2 fold changes between non-smokers and smokers of each tobacco type independently for each bacterial taxa identified^41^. Only taxa with significant differences between the study groups (p ≤ 0.05) were used to draw a heatmap using R version 4.0.1 (package: gplots; function: heatmap.2). Pheatmap package in R was used to draw the heatmaps of relative abundance of different genera and species in each sample.

Vegan package in R was used to calculate the estimates of alpha diversity (within sample diversity). The following diversity, richness, and evenness measures were used: Shannon and simpson diversity indices, Chao1-type estimator (diversity from abundance data), ACE (Abundance-based Coverage Estimator), S. OBS (Observed Species for counts of unique OTUs in each sample), and the Pielou index of species evenness, which refers to how equally abundant species in an environment are^42^. For beta diversity (between groups comparison), two distance metrics were used to assess the dissimilarity of samples: Bray–Curtis and Jaccard Index. Bray–Curtis takes into account the relative abundance of each species. The Jaccard index is binary and measures dissimilarity based on the presence/absence of each species. Each distance matrix was visualized through principal coordinates analysis (PCoA). For beta diversity, analysis was done using Partek Genomics Suite software, version 7.0 (Copyright 2020; Partek Inc., St. Louis, MO, USA).

## Results

Descriptive statistics are shown in Table 1. The majority of participants were male (85%) and ranged in age from 18 to 62, with medwakh-only users being slightly younger. In total, 30 (75%) participants had no or mild periodontitis and 10 (25%) had moderate to severe periodontitis. Cigarette smokers reported smoking 2–20 (12.3 ± 7.4) cigarettes per day every day. Medwack smokers reported smoking 2–50 (15.9 ± 16.8) times per day every day. For shisha smokers, they smoked 1–3 (1.2 ± 0.6) times per day in 1–7 days per week (frequency of smoking for each tobacco type is shown in Table 1). There were no former smokers among these participants, and all were exclusive smokers of a single tobacco type.

**Table 1:** Demographics and periodontal status of the study participants subdivided into four groups based on smoking status.

	Smokers			Non-smoker	p value
	Cigarettes	Shisha	Medwakh		
Subjects (n)	10	10	10	10	–
**Gender**					
Male	9 (90%)	8 (80%)	10 (100%)	7 (70%)	0.463
Female	1 (10%)	2 (20%)	0 (0%)	3 (30%)	
Age (years)	31.9 ± 10.43	29.1 ± 12.05	24.1 ± 4.33	38.5 ± 13.6	0.051
Duration of smoking (years)	12.8 ± 7.5	6.2 ± 6.4	6.3 ± 4.05	0	0.038*
Frequency of smoking^a^	12.3 ± 7.4	1.2 ± 0.6^b^	15.9 ± 16.8^c^	0	–
PPD (mm)	2.74 ± 0.74	2.54 ± 0.33	2.84 ± 0.47	2.19 ± 0.66	0.089
CAL (mm)	1.5 ± 1.03	1.3 ± 1.25	0.96 ± 0.47	0.69 ± 0.35	0.18
**BOP (%)**					
0–25	2 (20%)	0 (0)	0 (0)	3 (30%)	0.138
> 25	8 (80%)	10 (100%)	10 (100%)	7 (70%)	
**Periodontitis**					
No/mild	6 (60%)	9 (90%)	6 (60%)	9 (90%)	0.187
Moderate/severe	4 (40%)	1 (10%)	4 (40%)	1 (10%)	

Values express n (%) or mean ± SD.

*PD* pocket depth, *CAL* clinical attachment loss, *BOP* bleeding on probing.

*Significant at p < 0.05, non-smoker vs any smoker.

^a^Cigarettes/day, times per day for shisha and medwakh. Statistical comparison was not done due to the difference in the units of measurement (number of cigarettes/day, times/day with number of heads for shisha, times/day with number of puffs for medwakh).

^b^For shisha: 0.8 ± 0.3 heads/time.

^c^For medwakh: 6.4 ± 8.5 puffs/time.

More patients who smoked cigarettes or medwakh had moderate to severe periodontitis (40% in each group), while fewer shisha users and non-smokers had moderate to severe periodontitis (10% in each of group). All medwakh and shisha smokers had BOP ≥ 25%, while 70% of non-smokers had BOP ≥ 25%. When non-smokers were compared to all smoking groups combined, non-smokers had significantly lower PPD (p = 0.028) and CAL (p = 0.041) values. Inter-group comparisons showed that PPD was significantly higher (p = 0.049) among medwakh users when compared to non-smokers. There was a significant positive correlation between CAL and smoking duration, regardless of the type (correlation coefficient 0.354, p = 0.025; data not shown).

### Microbiome analysis

**Figure 1:**
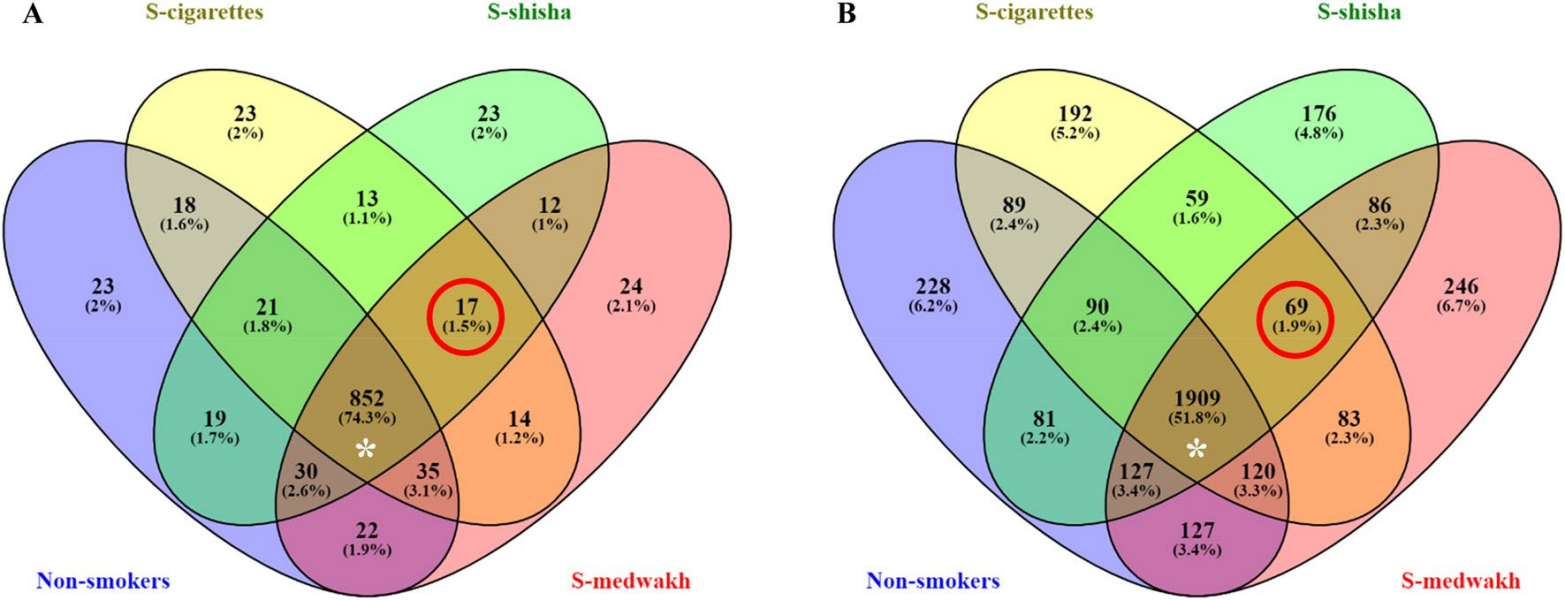
Venn diagram of exclusive and shared taxonomically unique microbiota ((**A**) genus, and (**B**) species) detected in subgingival plaque samples from non-smokers and smokers of cigarettes, medwakh, or shisha. Red circles indicate genera or species exclusively found in smoking groups (but not in non-smokers). Asterisk (*) represents the genera and species shared among all four groups.

A total of 37 phyla, 1146 genera, and 3682 species were detected. All phyla were found in at least one sample from each group of smokers and non-smokers, except *Lentisphaerae,* which was absent from all smokers of cigarettes. Two Venn diagrams (Fig. 1) were generated to illustrate the shared genera and species among the microbial communities of the four study groups (regardless of relative abundance). We found 852 (74.3%) common genera and 1909 (51.8%) common species, representing the core microbiota community (Fig. 1, asterisk annotated). About 2% of total genera and 4.8–6.7% of total species were exclusively present in one study group, but these genera and species were detected at a very low abundance. Moreover, 17 (1.5%) genera and 69 (1.9%) species were exclusively found in all 3 smoker groups but were absent among non-smokers (encircled in Fig. 1A,B), but again, these genera and species were detected at a very low abundance (Supplementary Tables S1, S2).

### Relative abundance

#### Phyla

**Figure 2:**
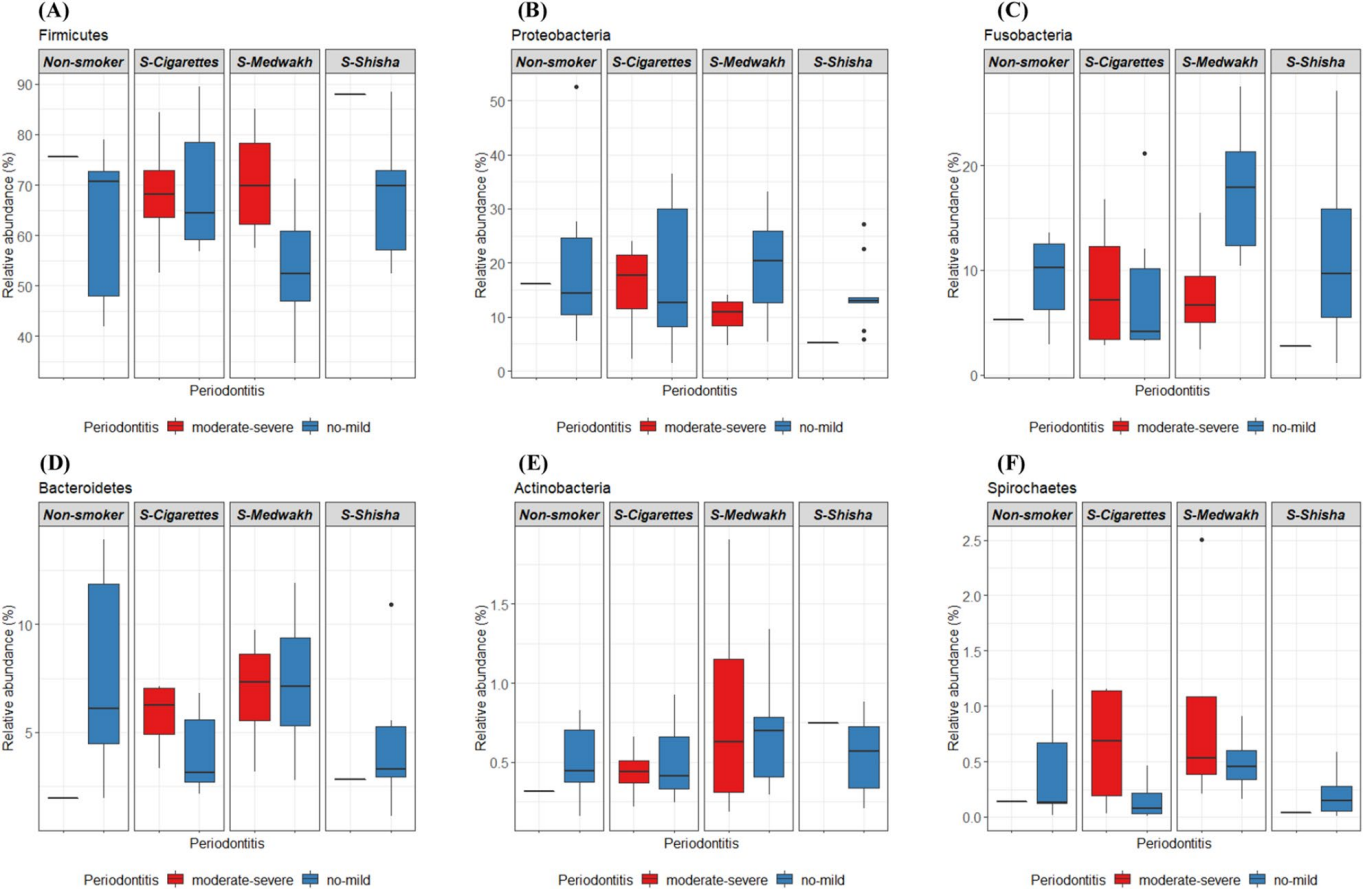
Comparison of the relative abundance (%) of the major phyla detected in subgingival plaque samples from non-smokers and smokers of cigarettes, medwakh and shisha with different stages of periodontal disease. Box plots show Q1-median-Q3 with range. Back dots are outlier values.

*Firmicutes* was the most abundant phyla in non-smokers (63.93 ± 13.75%), smokers of cigarettes (68.9 ± 12.62%), medwakh (60.22 ± 15.0%) and shisha (70.48 ± 13.06%). Seven major phyla (*Firmicutes, Proteobacteria, Fusobacteria, Bacteroidetes, Candidatus saccharibacteria, Actinobacteria,* and *Spirochaetes*) were detected at ≥ 1% abundance in at least one sample. A list of phyla (grouped based on relative abundance) are shown in Supplementary Fig. S1. A comparison of the relative abundances of phyla detected in the samples grouped by smoking type and periodontal disease severity is summarized in Fig. 2.

Briefly, shisha smokers had significantly less *Bacteroidetes* and *Spirochaetes* than medwakh smokers (Fig. 2D,F). Generally, there was no significant difference in the relative abundance of phyla in patients with no or mild periodontitis compared to patients with moderate or severe periodontitis. However, inter-group comparisons by smoking status and periodontitis showed there was significantly more *Fusobacteria* in medwakh smokers with no or mild periodontitis compared to those with moderate or severe periodontitis (Fig. 2C). In medwakh smokers, the relative abundance of *Firmicutes* was decreased (Fig. 2A) and *Proteobacteria* was increased (Fig. 2B) in patients with no or mild periodontitis compared to those with moderate to severe periodontitis, but the difference was not significant. In patients with no or mild periodontitis, the relative abundance of *Fusobacteria* was highest in medwakh smokers compared to non-smokers (Fig. 2C), and *Spirochaetes* were significantly increased in medwakh smokers compared to both cigarette and shisha smokers (Fig. 2F). In contrast, patients who smoked cigarettes, medwakh, or shisha and who had moderate or severe periodontitis did not have any significant difference in the relative abundance of all phyla, as compared to non-smokers (Fig. 2A–F).

#### Genera

**Figure 3:**
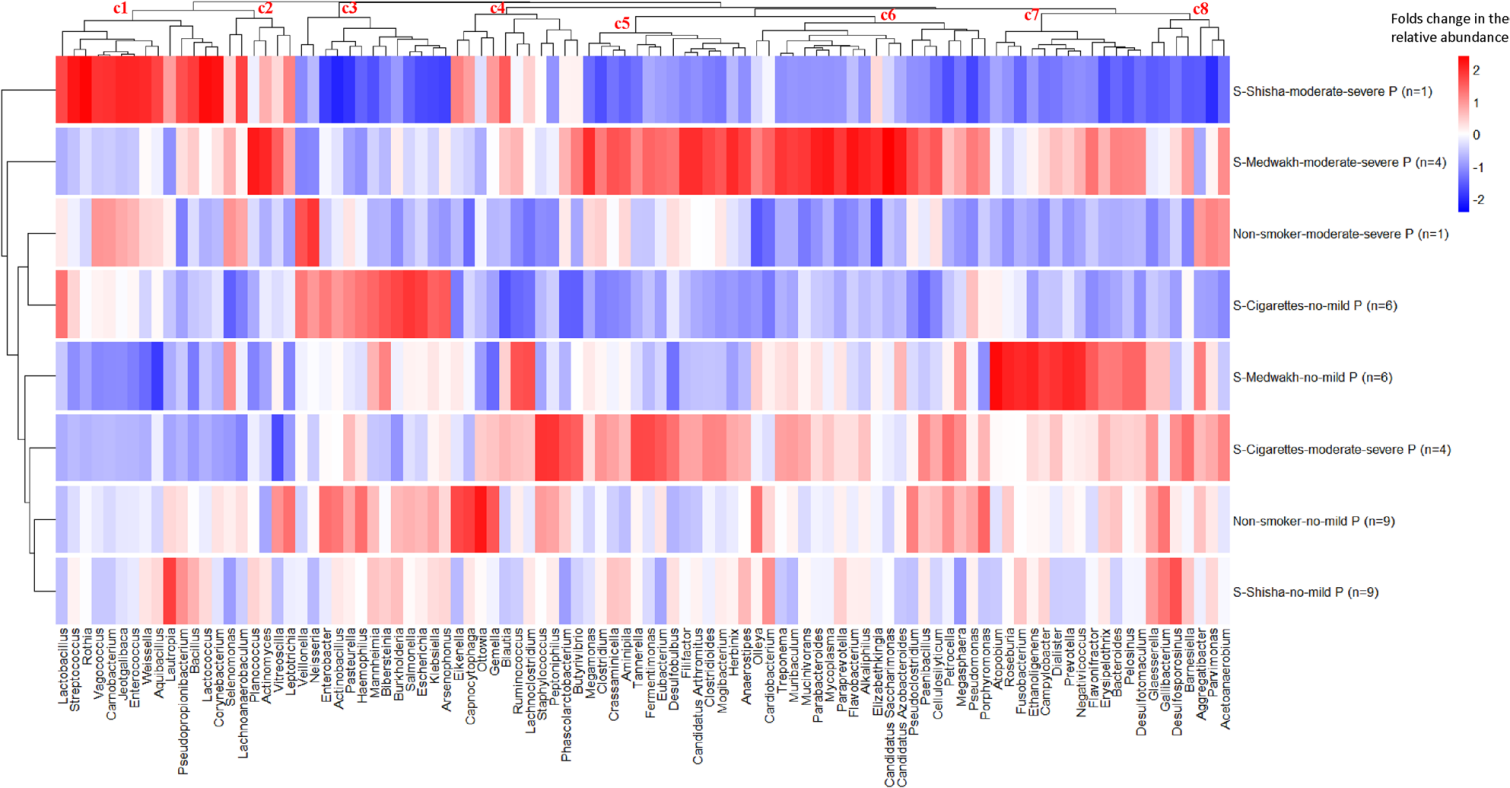
Distribution of different genera (relative abundance > 0.01%) grouped by smoking status and periodontal condition. Relative abundances of genera were ranked based on group counts (scaled across columns). Red color indicates high abundance in a particular group and blue color indicates low abundance. Dendrograms show clustering based on the relative abundance of different genera (left dendrogram: group clustering; top dendrogram: clustering of genera based on abundances in all the groups).

A total of 1146 genera were detected; however, only 98 genera had a relative abundance ≥ 0.1% in at least one sample. The top 10 genera detected were: *Streptococcus, Veillonella, Fusobacterium, Filifactor, Parvimonas, Campylobacter, Selenomonas, Gemella, Bacillus, Haemophilus,* and *Neisseria. Streptococcus* was the most common genus in the samples from all the study groups (26.78 ± 14.89% of total abundance). Figure 3 shows the dendrogram of genera relative abundance, grouped by smoking status and periodontal condition, with 8 unique clusters with different abundance among the study groups (c1–c8). Medwakh smokers with moderate to severe periodontitis had more genera with higher relative abundance compared to all the other groups (Fig. 3c2,c5–c8; red bars).

**Figure 4:**
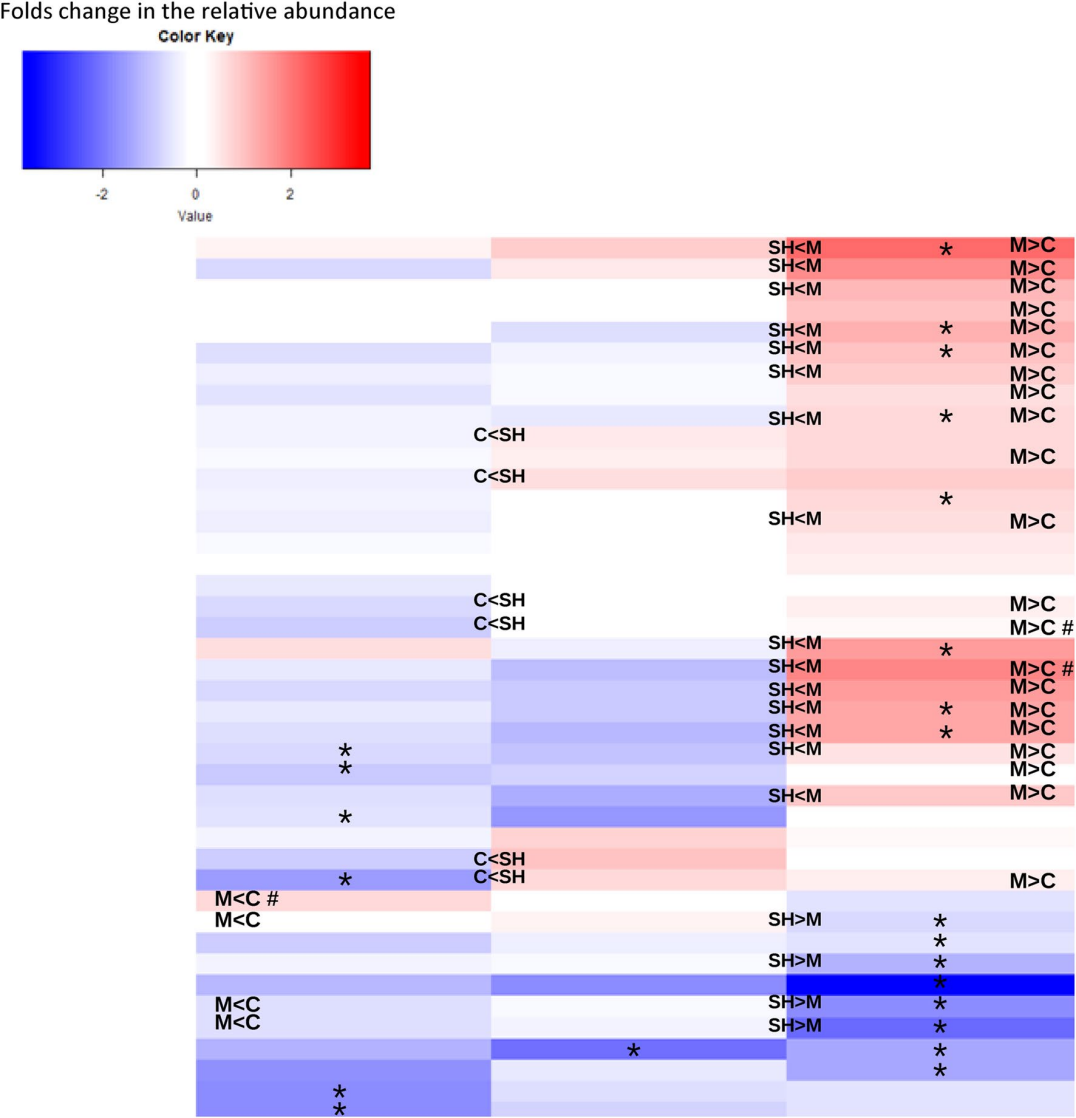
Significant log2 fold change of genera abundances in cigarette, shisha, or medwakh smoking groups compared to non-smokers (log2 tobacco vs log2 non-smokers). Red indicates an increase and blue indicates a decrease in the relative abundance of each genus compared to non-smokers. *Significant difference between each tobacco group and non-smokers; ^#^significant difference exclusively in patients with severe periodontitis; *M* medwakh smokers, *C* cigarette smokers, *SH* shisha smokers.

Statistical comparisons of the relative abundance of genera by study group revealed significant differences in 42 genera. Figure 4 demonstrates the relative difference in the abundance of each genus in cigarette, shisha, and medwakh groups compared to non-smokers (log2 fold changes are shown). Notably, more genera were enriched (expressed in higher quantities) in medwakh smokers compared to non-smokers, with a statistically significant difference found in *Desulfobulbus, Desulfotomaculum, Dialister, Flavonifractor*, and *Negativicoccus. Prevotella, Atopobium,* and *Fusobacterium* that were detected at significantly higher abundance in medwakh smoking patients with no-mild periodontitis, compared to non-smokers with the same periodontal condition. One the other hand, *Vagococcus, Ottowia, Porphyromonas, Gemella, Jeotgalibaca,* and *Enterococcus* were detected at significantly lower abundance in medwakh smokers compared to non-smokers. *Corynebacterium* and *Streptococcus* were detected at significantly lower abundance in medwakh smokers only in patients with no-mild periodontitis compared to non-smokers with the same periodontal condition. *Olleya, Cardiobacterium, Bacteroides, Muribaculum, Gallibacterium,* and *Eikenella* were significantly depleted in cigarette smokers compared to non-smokers (Fig. 4), especially in patients with no or mild periodontitis. *Porphyromonas* was significantly depleted in shisha smokers compared to non-smokers (Fig. 4), while the remaining genera were not significantly different from non-smokers even after considering the patients’ periodontal condition. Additionally, significant variations in the relative abundance of genera between smoking groups (cigarette, shisha, medwakh) were also found. Notably, medwakh smokers exhibited significantly higher relative abundance of many genera compared to cigarette and shisha smokers (shown in Fig. 4).

When subjects with no or mild periodontal disease are compared to those with moderate or severe periodontal disease in each of the four study groups, few significant differences were found. Medwakh smokers with no or mild periodontal disease had significantly more *Fusobacterium*, and significantly less *Roseburia* and *Desulfobulbus,* than those with a moderate to severe form of the disease. *Tannerella, Desulfosporosinus, Butyrivibrio, Blautiawas,* and *Lachnoclostridium* were significantly higher in cigarette smokers with moderate to severe periodontal disease compared to those with no or mild periodontal disease.

#### Species

**Figure 5:**
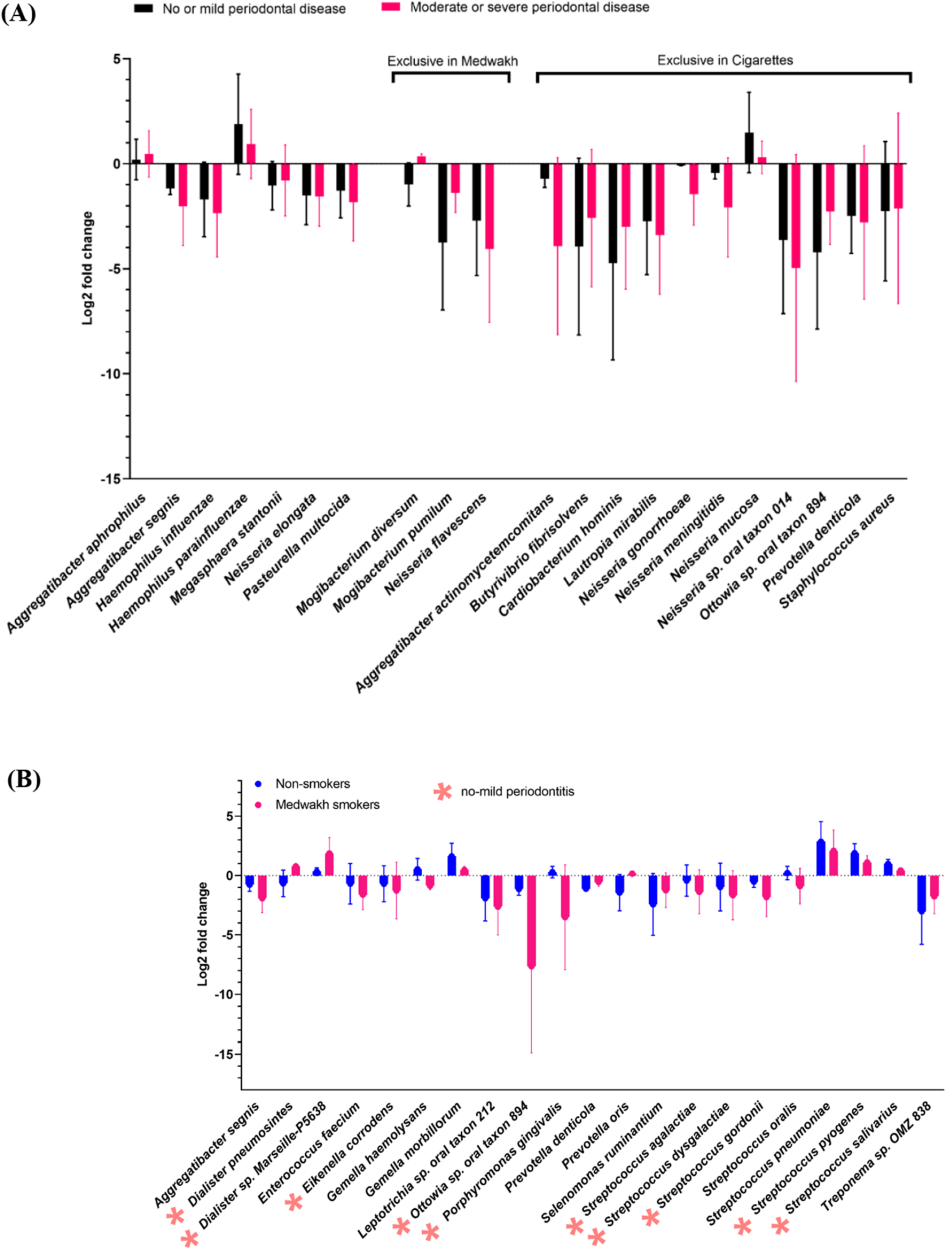

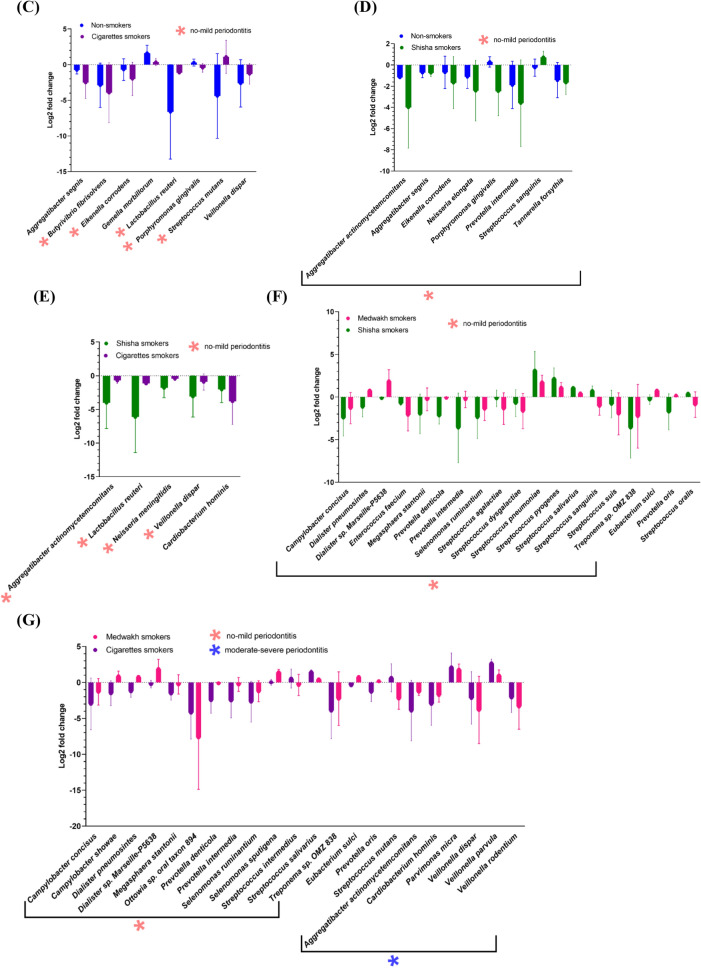
Comparison of the relative abundance (log 2 transformed ± SD) of species by (**A**) periodontal disease severity and (**B**–**G**) smoking status. Only significantly different species are shown. Species exclusively differentially abundant in patients with no or mild periodontal disease and patients with moderate or severe periodontal disease are marked with pink and blue asterisks, respectively (**B**–**G**).

A total of 3682 species were detected; however, only 82 species were detected at a relative abundance ≥ 1% in at least one sample and were used for further statistical analysis. Species with significant differences in their relative abundance by study groups are shown in Fig. 5.

Comparison of the relative abundance of different species in patients with no or mild periodontal disease and patients with moderate or severe periodontal disease indicated that 7 species were differentially abundant between the two groups (Fig. 5A). Three species were differentially abundant in medwakh smokers and 10 in cigarette smokers, in patients with no or mild periodontal disease compared to those with moderate or severe disease. Interestingly, two species that are well-known as periodontopathogens (*Aggregatibacter actinomycetemcomitans* and *Prevotella denticola*) were increased in cigarette smokers with no or mild periodontitis compared to those with moderate or severe disease. Species that are part of the normal healthy flora, such as *Lautropia mirabilis* and *Neisseria mucosa,* were significantly lower in cigarette smokers with moderate or severe periodontal disease compared to those with no or mild disease. No significant differences were found among shisha smokers or non-smokers by periodontal disease severity.

Several significant differences were found in the relative abundance of species between various smoking groups and non-smokers. Importantly, we identified significant differences in the relative abundance of important periodontopathogens, especially in samples collected from patients with no or mild periodontal disease. *Porphyromonas gingivalis, Eikenella corrodens* and *Streptococcus gordonii* were less abundant in medwakh smokers compared to non-smokers, while *Prevotella denticola* and *Treponema* sp. *OMZ 838* were more abundant in medwakh smokers compared to non-smokers (Fig. 5B). *Eikenella corrodens* and *Porphyromonas gingivalis* were less abundant in cigarette smokers compared to non-smokers, while *Streptococcus mutans* and *Veillonella dispar* were more abundant in cigarette smokers compared to non-smokers (Fig. 5C). *Aggregatibacter actinomycetemcomitans, Eikenella corrodens, Porphyromonas gingivalis* and *Prevotella intermedia* were less abundant in shisha smokers compared to non-smokers, while *Streptococcus sanguinis* and *Tannerella forsythia* were more abundant in shisha smokers compared to non-smokers (Fig. 5D). Intergroup comparisons of medwakh, shisha, and cigarette smokers also indicated significant differences in the relative abundance of various species, especially in samples collected from patients with no or mild periodontal disease (Fig. 5E–G). Periodontopathogens bacteria as *Veillonella dispar* was more in cigarettes than each of medwakh and shisha smokers, while *Aggregatibacter actinomycetemcomitans* was more in medwakh than cigarettes smokers, and more in cigarettes than shisha smokers. Additionally, three important pathogens (*Prevotella intermedia*, *Prevotella denticola* and *Treponema* sp. *OMZ 838*) were more in medwakh smokers than both of cigarettes and shisha smokers (Fig. 5F,G).

Among periodontopathogens, *Aggregatibacter actinomycetemcomitans and Veillonella dispar* were more in cigarettes smokers compared to shisha smokers (Fig. 5E). When medwakh smokers were compared to shisha smokers, multiple species of genus *Streptococcus,* were less in the former than the latter. Some of them, like *Streptococcus sanguinis,* are important cariogenic bacteria (Fig. 5F). In patients with moderate or severe periodontal disease, multiple species of the genus *Veillonella* (*Veillonella dispar, Veillonella parvula* and *Veillonella rodentium*) and *Parvimonas micra* were more in cigarettes than medwakh smokers, while *Aggregatibacter actinomycetemcomitans* and *Cardiobacterium hominis* were more in medwakh than cigarettes than smokers (Fig. 5G).

### Diversity

**Figure 6:**
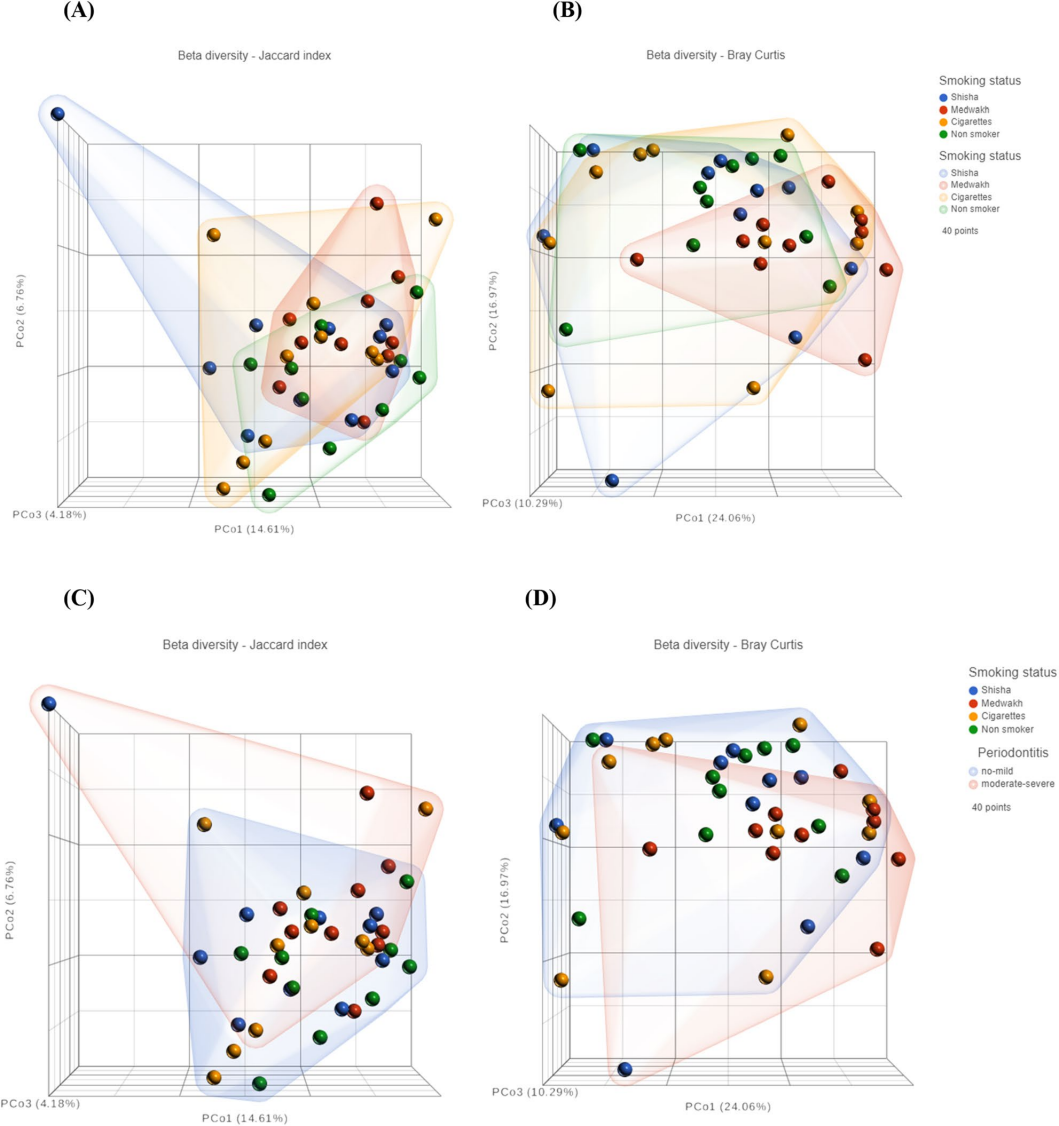

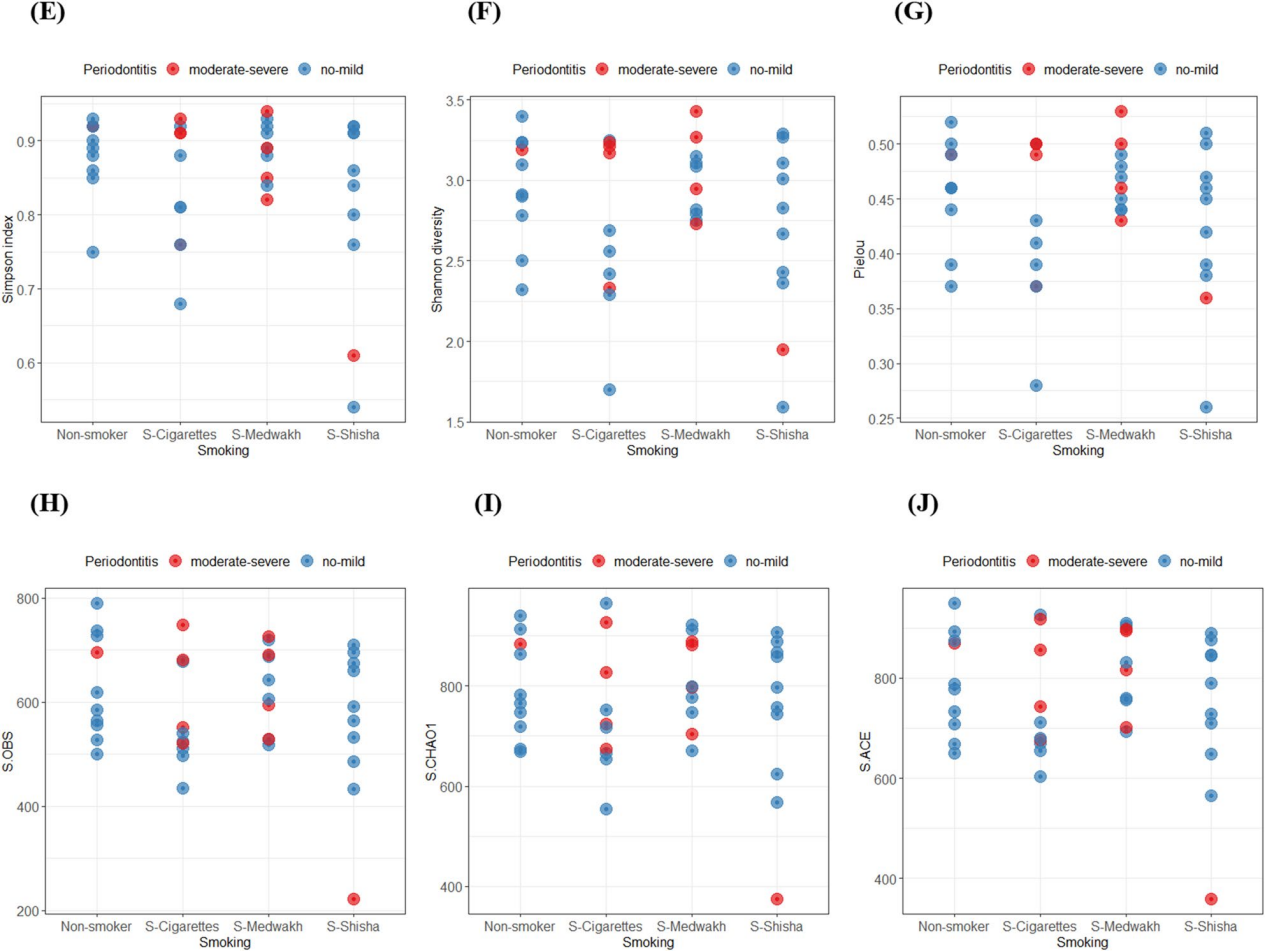
Beta diversity (**A**–**D**) and alpha diversity (**E**–**J**) of 40 subgingival plaque samples. For beta diversity, Jaccard index (**A**,**C**) and Bray–Curtis index (**B**,**D**) are shown. Samples were clustered based on principal coordinates analysis (PCoA). In (**A**,**B**), samples were highlighted by smoking group, and in (**C**,**D**), samples were highlighted based on periodontal condition. PCo1, PCo2 and PCo3 are shown in the figures. The percentage of variation explained by each principal coordinate is indicated on the axes. Each point represents a microbial community from one sample. For alpha diversity (**E**–**J**), samples were grouped based on both smoking status and periodontal condition.

Figure 6A,D shows beta diversity dissimilarity analysis (between groups comparison) using Bray–Curtis and Jaccard indices. Based on the PCoA analysis, the four smoking groups overlapped such that no group was unique in comparison to the others. However, a few individual samples were unique in that they did not fit into the PCoA overlap (Fig. 6A,B). When grouping by periodontal condition of the patients, all unique (non-overlapped) samples belonged to patients with moderate to severe periodontitis (Fig. 6C,D).

For alpha diversity (within sample diversity), different diversity measures were used, including Shannon and Simpson diversity indices, Chao1-type, ACE, OBS and Pielou index (Fig. 6E–J). In cases with no-mild periodontitis, we found that cigarette smokers had significantly less Pielou and OBS indices than non-smokers (p < 0.05). Furthermore, Simpson and ACE indices were significantly lower in cigarette smokers than medwakh smokers with no-mild periodontitis (p < 0.05).

## Discussion

This study provides a detailed insight into the composition of the subgingival microbiome in a small group of smokers of medwakh, shisha, and cigarettes with or without periodontal disease, compared to non-smoking individuals. A variety of significant differences in pathogenic bacterial abundance were found between the study groups, suggesting that smoking not only cigarettes, but also shisha and medwakh may increase risk for periodontal disease. Subgingival microbiome of smokers was altered even in subjects with no or mild periodontitis, in agreement with Karasneh et al*.*, who also found a significant increase in the prevalence of periodontopathogenic bacteria and depletion of beneficial bacteria in subgingival samples of smokers with healthy gingiva^12^.

Previous studies have shown that cigarette smokers have a higher risk of severe periodontitis than non-smokers^43^. Similarly, in our study we found that, compared to non-smokers, all smoking groups had significantly higher values of PPDs and CALs. In addition, CAL was positively correlated with duration of smoking, regardless of the type. Even though we had a smaller sample size, this finding is in accordance with epidemiologic studies that found increased risk of periodontitis in smokers proportional to the duration and rate of smoking^44^. Among our patients, 40% of cigarette and medwakh smokers had moderate to severe periodontitis, while 10% of shisha smokers had severe periodontitis. This may be explained by less frequent exposure to shisha smoke, since it is considered a social and recreational activity, as compared to highly frequent use of cigarettes and medwakh throughout the day.

Previous studies evaluating the effect of smoking on the subgingival plaque microbiome have found variable results. Some studies concluded that smoking did not have any significant effect on the subgingival microbiome^45,46^, while others found increased periodontitis and periodontal pathogen counts in smokers^47,48^. The conflicting findings in these studies might be partly explained by the sensitivity and specificity of the microbiological methods used, as different culture-based and molecular methods yield inconsistent results in determining the relative abundance of periodontal pathogens. In this study, we used the latest next generation 16S rRNA sequencing technology by Oxford nanopore to explore the subgingival microbiome of smokers of different tobacco types. We observed a very high number of microbes in our samples; core microbiome analysis indicated that 852 (74.3%) common genera and 1909 (51.8%) common species were shared between the 4 study groups. The relative abundance of genera and species was variable and was influenced by both smoking and periodontal condition.

In many previous studies^19,29,49^, periodontitis was the only factor investigated in relation to the composition of subgingival plaque, and other factors such as smoking were not considered, a clear limitation the current study addresses. We found no significant difference in the relative abundance of phyla when all patients were grouped based on their periodontal condition regardless of smoking status; however, some phyla were significantly different among smokers of different tobacco types, especially in medwakh smokers. We found that the relative abundance of *Firmicutes* was decreased and *Proteobacteria* was increased in medwakh smokers with no or mild periodontitis compared to those with moderate to severe periodontitis, but the difference was not significant. *Fusobacteria* in medwakh smokers was significantly more abundant in patients with no or mild periodontitis than those with moderate or severe periodontitis.

Other studies have focused on how the oral microbiome changes in response to smoking, without considering the periodontal condition of subjects. In the USA, one study reported a significant depletion of *Proteobacteria*, and enrichment of *Firmicutes* and *Actinobacteria*, in oral wash samples of cigarette smokers compared to non-smokers^50^. We did not find such alteration in our study population of cigarette smokers. Another study in the UAE reported depletion of the phyla *Proteobacteria* and *Bacteroidetes* and enrichment of the phyla *Actinobacteria* in oral wash samples of both medwakh and cigarettes smokers^41^. This study also reported depletion of the phyla *Cyanobacteria* and *SR1* in shisha smokers compared to non-smokers. In contrast, we found that *Bacteroidetes* were enriched in subgingival plaque samples of medwakh smokers compared to shisha smokers. However, the results are not directly comparable as we used plaque samples rather than mouthwash^50,51^. A previous study reported that pooled subgingival plaques collected by paper points from the deepest pocket of each quadrant in the mouth (as we did in this study) were superior to mouthrinse samples. The detection frequency of key periopathogens was significantly higher in subgingival plaques than mouthrinse samples in patients with periodontitis^33^.

Microbial profiling based on genera relative abundances revealed clear differences among the study groups (Fig. 3). Medwakh smokers, especially those with moderate or severe periodontitis expressed more genera with higher abundance compared to non-smokers and smokers of other tobacco types (Figs. 3, 4). Compared to non-smokers, *Corynebacterium, Streptococcus*, *Vagococcus, Ottowia, Gemella, Jeotgalibaca* and *Enterococcus* were significantly depleted in medwakh smokers, in contrast to the report of Valles et al. which indicated the depletion of *Actinobacillus, Lautropia*, and *Porphyromonas* among medwakh smokers. *Olleya, Cardiobacterium, Bacteroides, Muribaculum, Gallibacterium* and *Eikenella* were significantly depleted in cigarette smokers compared to non-smokers. In contrast, Valles et al*.* 2018 reported depletion of the genera *Neisseria, Eikenella, Aggregatibacter, Actinobacillus, Haemophilus* and *Lautropia*, *Fusobacterium* and *Leptotrichia* in mouthwash samples of cigarette smoker compared to non-smokers; thus, *Eikenella* was the only shared depleted genus between this study and Valles et al*.* 2018. In this study, *Porphyromonas* was significantly depleted in subgingival samples of both cigarette and shisha smokers compared to non-smokers, in agreement with the findings of Valles et al*.* 2018 who reported that *Porphyromonas* was significantly depleted in mouthwash samples of both cigarette and medwakh smokers, but not in shisha smokers^41^.

*Streptococcus*, one of the most important genera in the oral cavity, was the most abundant genus in all the study groups. Previous studies have reported high abundance of *Streptococcus* in healthy cigarette smokers. *Streptococcii* are facultative or obligate anaerobes and are generally acid tolerant, thus, they can tolerate the smoking oral environment^50^. In our study, the relative abundance of *Streptococcus* was significantly decreased in subgingival plaque samples of medwakh smokers compared to non-smokers, cigarette smokers, and shisha smokers with or without mild periodontal disease. To the best of our knowledge, this is the first report documenting the depletion of subgingival *Streptococcus* in medwakh smokers. *Streptococcus* is abundant in health-associated biofilms, but its levels decrease in diseased patients^52^. Previous studies have shown that early stage periodontal disease (gingivitis) is preceded by a decrease in the abundance of early colonizers, such as *Streptococcus* and *Veillonella,* in the subgingival biofilms of cigarette smokers^11,53^. Microbial profiles of smokers with moderate to severe chronic periodontitis have found lower levels of *Veillonella, Neisseria, and Streptococcus* and greater abundance of *Parvimonas, Fusobacterium, Campylobacter, Bacteroides,* and *Treponema*^49^. Notably, we fund *Parvimonas micra* at significantly higher abundance in subgingival samples of cigarette smokers with moderate to severe periodontitis in this study. Also, *Veillonella dispar* was significantly more abundant in cigarettes than each of medwakh and shisha smokers.

It has previously been reported that smoking increases the abundance of periodontal pathogens such as *Fusobacterium* and *Bacteroides*, as well as the severity of periodontitis. In this study, medwakh smokers with no or mild periodontal disease had significantly more *Fusobacterium* in subgingival plaque samples than those with a moderate to severe form of the disease. It was also elevated in cigarette and shisha smokers, but the difference was not statistically significant. *Fusobacterium* has been recognized as one of the major genera of subgingival bacterial community shift induced by smoking^29^. In particular, *F. nucleatum* is considered a “bridging species” that facilitates the formation of a pathogenic subgingival biofilm, thus contributing to the progression and severity of periodontal disease^11^.

Other bacteria that are consistently associated with periodontal disease include *Parvimonas, Treponema, Filifactor*, and *Bacteroides*^54,55^. In our study, almost all of these genera were significantly more abundant in medwakh smokers, even those with mild periodontal disease. For *Bacteroides,* the relative abundance was significantly higher in subgingival samples of medwakh smokers and was greater than that found in cigarette and shisha smokers with or without mild periodontal disease. These findings suggest that smoking medwakh may enrich *Bacteroides*, even in patients with no or mild periodontal disease, potentially increasing risk of progression to more severe disease^49^.

At the species level, we focused on the analysis of key periodontal pathogens. *Prevotella denticola* and *Treponema* sp. OMZ 838 were more abundant in medwakh smokers compared to non-smokers, and to cigarette smokers with no or mild periodontal disease. Previous research has found increased periodontal pathogens in the oral microbiome of smokers. For example, a study in Finland found a higher prevalence of *P. intermedia*, *T. forsythia* and *T. denticola* among cigarette smokers compared to non-smokers^56^. Our results are consistent with that study. We found that *T. forsythia* was elevated in shisha smokers compared to non-smokers, while *P. intermedia* was increased in medwakh smokers compared to both cigarette and shisha smokers. Compared to non-smokers, we found some cariogenic bacteria at higher abundance, including *Streptococcus sanguinis* and *Streptococcus mutans*, in shisha smokers and cigarette smokers, respectively.

In our study, *Aggregatibacter actinomycetemcomitans* was more abundant in medwakh than cigarettes smokers, and more abundant in cigarettes than shisha smokers and non-smokers. Recently, *Aggregatibacter actinomycetemcomitans* has been identified as one of the pathogens detected at a high quantity in subgingival samples of cigarette-smokers and users of electronic nicotine delivery systems (ENDS) using used culture-based methods^57^. The latter study, also reported *P. gingivalis* at significantly higher quantity among cigarette-smokers and ENDS-users than non-smokers with periodontitis, whereas, *T. denticola* was more prevalent in cigarette-smokers, ENDS-users and non-smokers with periodontitis compared with non-smokers without periodontitis.

*Porphyromonas gingivalis* and *Streptococcus gordonii* were less abundant in medwakh smokers compared to non-smokers. *S. gordonii,* is an early colonizer in the oral cavity that interacts with *P. gingivalis*. Research found that exposure to tobacco in cigarettes smokers has promoted the formation of a dual species biofilm composed of both *S. gordonii* with *P. gingivalis*^58^. Other in vitro studies have shown that treatment with nicotine at concentrations similar to those present in saliva of smokers can enhance *S. gordonii* planktonic cell growth, thus may contribute to *S. gordonii-P. gingivalis* biofilm formation; nevertheless, the role of nicotine on *P. gingivalis* growth is not yet clear^59^. It was found that a single exposure to nicotine treatment was able to inhibit the growth of *P. gingivalis* in a dose-dependent manner, but its growth rate increased after multiple repeated exposure to nicotine. Another study reported higher colonization of *Aggregatibacter actinomycetemcomitans*, but less colonization of *P. gingivalis* after the inoculation of primary epithelial cell monolayers with nicotine and cotinine at a concentration of 1 mg/ml in a time-dependent manner^60^. It is obvious that the growth of a single microbe is influenced by the presence of other bacteria within the same community. Thus, it is possible that dysbiosis in the subgingival microbiome of smokers affects the overall microbial community; thus, some species were enriched while others were depleted.

Research suggests that a more diverse bacterial community represents a more stable and healthy ecosystem^61^; thus, reduction of oral microbial diversity has been considered a predisposing factor for several diseases^11^. Several studies found that bacterial diversity estimates including both richness and evenness were higher in patients with chronic periodontitis than in periodontally healthy subjects^28,61,62^. In contrast, other studies have found that microbial diversity and species richness do not differ between periodontitis and healthy control samples^63,64^. Our findings support the latter conclusion; we found no significant differences in microbial diversity and richness estimates between patients with no-mild periodontitis and those with moderate or severe periodontitis.

Smoking has also been associated with microbial community diversity. Bizzarro et al*.* found that bacterial diversity in periodontal patients decreased with smoking. In our study, the effect of smoking was more obvious in patients with no-mild periodontitis, especially in cigarette smokers who had significantly less evenness (Pielou index) and richness (OBS index) of the microbial community compared to non-smokers. Furthermore, diversity and richness (Simpson and ACE indices) were significantly decreased in cigarette smokers than medwakh smokers with no-mild periodontitis. These findings suggest that periodontal ecology is affected by smoking, with a higher toxic effect from cigarettes. This may be related to the duration of smoking, which was significantly longer in cigarette smokers than medwakh smokers (Table 1).

In conclusion, using 16S rRNA sequencing, we found altered and markedly heterogenous subgingival microbial communities in smokers of cigarettes, medwakh, and shisha. In agreement with other investigators^19,30,65^, our findings suggest that periodontal microbiome dysbiosis is promoted by smoking of not only cigarettes, but also medwakh and shisha, and is associated with periodontitis.

This study was limited by the small sample size. There was a paucity of participants who were exclusive smokers of a single tobacco type and fulfilled the inclusion and exclusion criteria. Nevertheless, this pilot study provides essential new knowledge about the impact of smoking of different tobacco types on oral health. The results from this study warrant the design of large-scale prospective studies to investigate use of the microbiome profile for diagnosis and risk assessment of periodontitis^51^. We recommend further studies on the periodontal microbiome to elucidate the impact of tobacco use cessation on periodontal disease progression and oral microbiome composition, as well as evaluation of other interventional therapies.

## Supplementary Information

Supplementary Information 1.

Supplementary Information 2.

Supplementary Information 3.

## Data Availability

The 16S rRNA sequencing data that support the findings of this study have been deposited in the Sequence Read Archive (PRJNA658726), along with demographic metadata, to be released upon publication. Additional data on the study participants are available from the corresponding author upon reasonable request.
