# Publisher Correction: The impact of smoking different tobacco types on the subgingival microbiome and periodontal health: a pilot study

**DOI:** 10.1038/s41598-021-89261-w

**Published:** 2021-05-03

**Authors:** Sausan Al Kawas, Farah Al-Marzooq, Betul Rahman, Jenni A. Shearston, Hiba Saad, Dalenda Benzina, Michael Weitzman

**Affiliations:** 1 Department of Oral and Craniofacial Health Sciences, College of Dental Medicine, University of Sharjah, Sharjah, UAE; 2 Sharjah Institute for Medical Research, University of Sharjah, Sharjah, UAE; 3 Department of Medical Microbiology and Immunology, College of Medicine and Health Sciences, UAE University, P.O. Box 15551, Al Ain, UAE; 4 Department of Preventive and Restorative Dentistry, College of Dental Medicine, University of Sharjah, Sharjah, UAE; 5 Department of Pediatrics, School of Medicine, New York University, New York, USA; 6 New York University Abu Dhabi, Al Ain, UAE; 7 Department of Environmental Health Sciences, Mailman School of Public Health, Columbia University, New York, USA; 8 Department of Environmental Medicine, School of Medicine, New York University, New York, USA; 9 College of Global Public Health, New York University, New York, USA

Correction to: *Scientific Reports* 10.1038/s41598-020-80937-3, published online 13 January 2021

This Article contains errors in Figure 4 where the horizontal and vertical axis labels are missing. The correct Figure 4 appears below as Figure 1.

**Figure 1:**
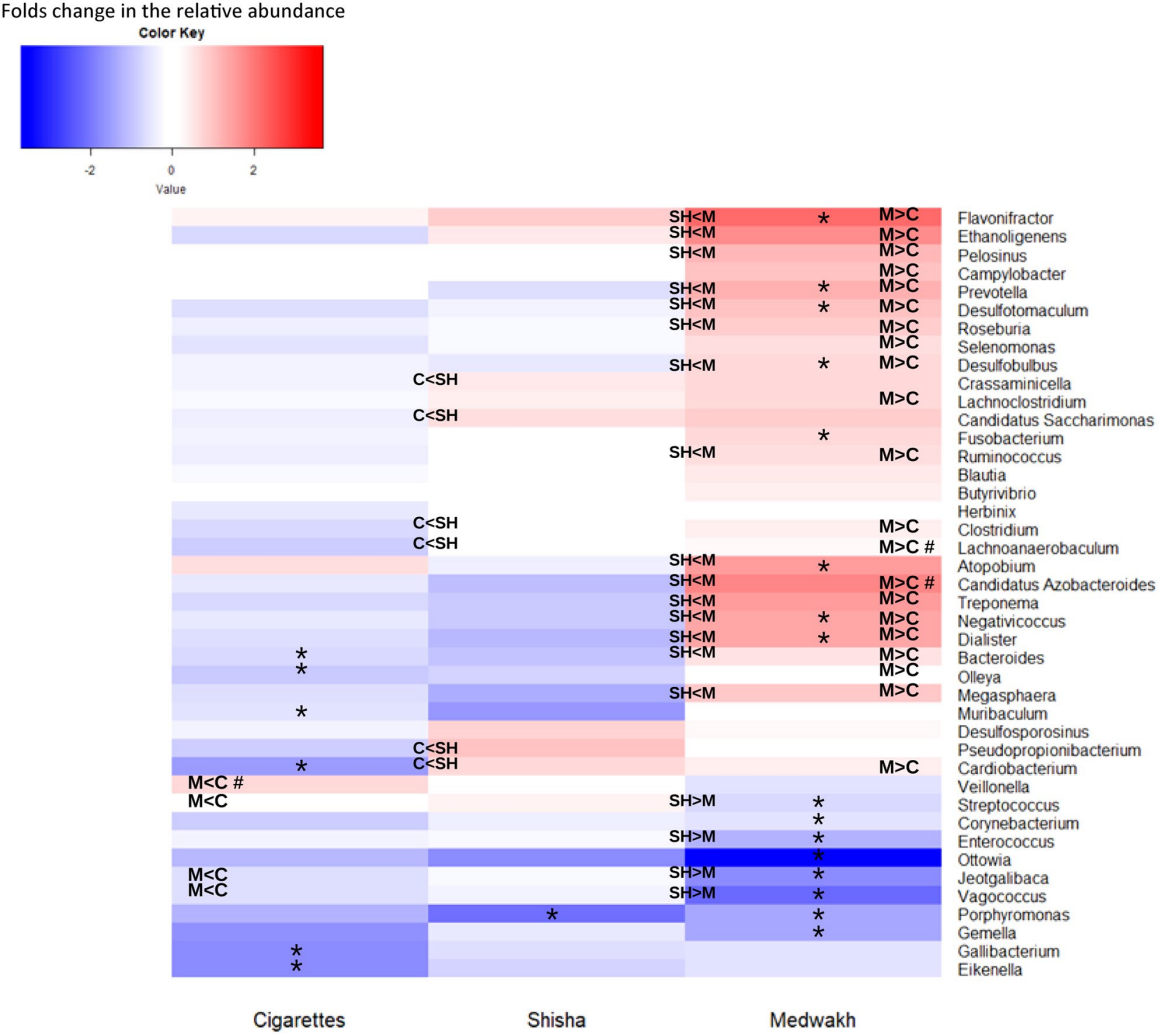
Significant log2 fold change of genera abundances in cigarette, shisha, or medwakh smoking groups compared to non-smokers (log2 tobacco vs log2 non-smokers). Red indicates an increase and blue indicates a decrease in the relative abundance of each genus compared to non-smokers. *Significant difference between each tobacco group and non-smokers; #significant difference exclusively in patients with severe periodontitis; M medwakh smokers, C cigarette smokers, SH shisha smokers.

