# Supplementary material for: The impact of smoking different tobacco types on the subgingival microbiome and periodontal health: a pilot study
Source: Sci Rep. 2021 Jan 13;11:1113. doi: 10.1038/s41598-020-80937-3 (PMC7806658; doi:10.1038/s41598-020-80937-3)
Supplement: Supplementary file 1 — Supplementary Information 1. [file 41598_2020_80937_MOESM1_ESM.docx]

**Supplementary files**

**The Impact of Smoking Different Tobacco Types on the Subgingival Microbiome and Periodontal Health: A pilot study**

Sausan Al Kawas^1,2^, Farah Al-Marzooq^3^*, Betul Rahman^2,4^, Jenni A. Shearston^5,6,7^, Hiba Saad^1^, Dalenda Benzina^1^, Michael Weitzman^5,6,8,9^

^1.^ Department of oral and Craniofacial Health Sciences, College of Dental Medicine, University of Sharjah, United Arab Emirates

^2.^ Sharjah institute for medical research, University of Sharjah, Sharjah, United Arab Emirates

^3.^ Department of Medical Microbiology and Immunology, College of Medicine and Health Sciences, UAE University, Al Ain, United Arab Emirates

^4.^ Department of Preventive and Restorative Dentistry, College of Dental Medicine, University of Sharjah, United Arab Emirates

^5.^ Department of Pediatrics, School of Medicine, New York University, USA

^6.^ New York University Abu Dhabi, United Arab Emirates

^7.^ Department of Environmental Health Sciences, Mailman School of Public Health, Columbia University, USA

^8.^ Department of Environmental Medicine, School of Medicine, New York University, USA

^9.^ College of Global Public Health, New York University, USA


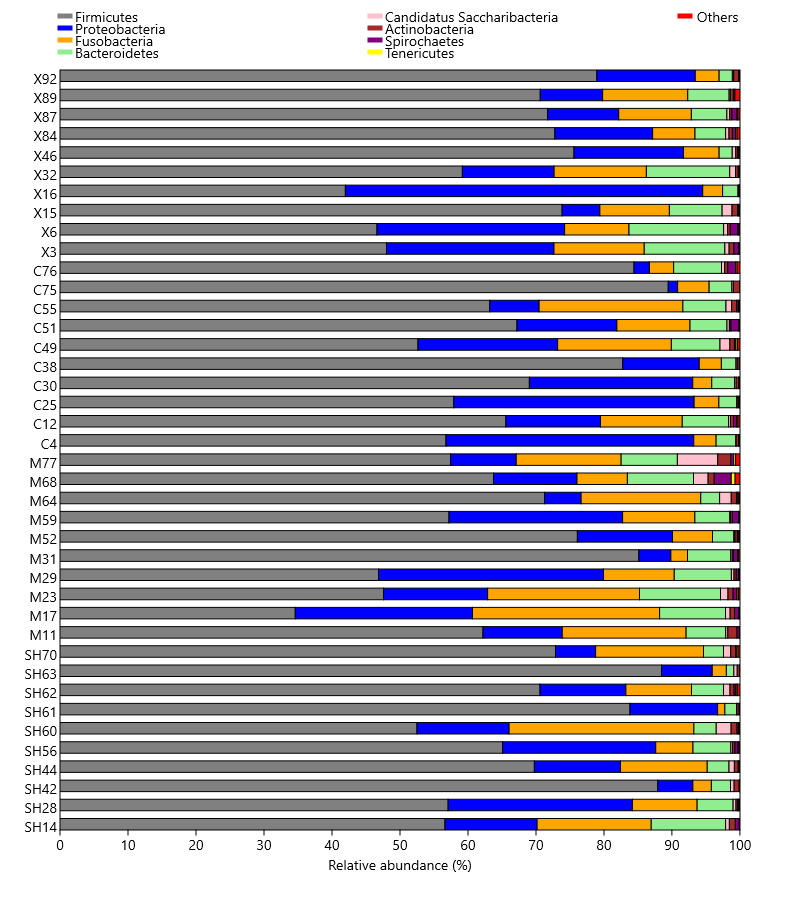


**Figure S1. The relative abundance (%) of phyla detected in 40 subgingival plaque samples**

**Table S1. Genera detected in different study groups (one, two, three or all the four study groups) identified by Venn diagram (Figure 1 A)**

**Attached as excel file with multiple sheets**

**Table S2. Species detected in different study groups (one, two, three or all the four study groups) identified by Venn diagram (Figure 1B)**

**Attached as excel file with multiple sheets**
